# Couples Dealing With Pediatric Blood Cancer: A Study on the Role of Dyadic Coping

**DOI:** 10.3389/fpsyg.2019.00402

**Published:** 2019-02-27

**Authors:** Marieke Van Schoors, Tom Loeys, Liesbet Goubert, Geertrui Berghmans, Britt Ooms, Jurgen Lemiere, Koenraad Norga, Lesley Liliane Verhofstadt

**Affiliations:** ^1^Department of Experimental Clinical and Health Psychology, Ghent University, Ghent, Belgium; ^2^Department of Data Analysis, Ghent University, Ghent, Belgium; ^3^Department of Pediatric Hemato-Oncology and Stem Cell Transplantation Ghent, Ghent University Hospital, Ghent, Belgium; ^4^Department of Pediatric Hemato-Oncology and Immunology, University Hospital Brussels, Brussels, Belgium; ^5^Department of Pediatric Hemato-Oncology, University Hospital Leuven, Leuven, Belgium; ^6^KU Leuven, Leuven, Belgium; ^7^Department of Pediatric Oncology, Antwerp University Hospital, Antwerp, Belgium; ^8^University of Antwerp, Antwerp, Belgium

**Keywords:** couples, intimate relationships, pediatric cancer, dyadic coping, individual adjustment, relationship adjustment

## Abstract

**Objective:** Pediatric cancer is a life-threatening disease that poses significant challenges to the ill child and his/her parents. Among the studies investigating risk and protective factors for the individual and relationship adjustment of parents being confronted with pediatric cancer, couple factors – such as dyadic coping – gained little research attention. Therefore, the aim of the current study was to explore the association between dyadic coping and individual/relationship outcomes of parents in the context of pediatric cancer.

**Methods:** Participants were 59 couples of children diagnosed with leukemia or Non-Hodgkin lymphoma. Time since diagnosis varied from diagnosis to 20 months. Both parents completed the DCI-short, DASS21, PIP, and MMQ.

**Results:** Positive dyadic coping (i.e., supportive and common dyadic coping) and negative dyadic coping proved to be related to individual and relational outcomes of parents facing cancer in their child. In addition, while men and women reported to be equally satisfied with their partner and their sexual relationship, women reported higher levels of individual maladjustment.

**Conclusion:** Our findings led to the conclusion that dyadic coping is important for both individual as well as relationship outcomes of parents when facing a diagnosis of cancer in their child. When meeting with families, both partners should be invited as a unit in order to best capture couple level experiences. Also, clinicians should be sensitive to relational and sexual issues besides individual issues, taking into account evidence-based standards for psychosocial care in pediatric oncology.

## Introduction

Pediatric cancer is an unpredictable and uncontrollable stressor that puts the diagnosed child at risk for adjustment difficulties (Alderfer and Kazak, 2006). There are a number of pediatric cancers, with blood cancer, including leukemia and lymphoma, as the most common type. Leukemia and lymphoma account for about 30 and 8% of all cancers in children, respectively (American Cancer Society, 2016). Due to advances in chemotherapy and stem cell transplantation, long-term survival of children with blood cancer can be achieved (Silverman and Weinstein, 1997). However, although many function well, some children with blood cancer (Rao et al., 1992; van der Does-van den Berg et al., 1995) or pediatric cancer in general (Kazak et al., 2001; Kestler and LoBiondo-Wood, 2012) experience social or emotional problems during or after treatment. In addition, the impact of a pediatric cancer diagnosis on the ill child’s parents is undeniable. Every child is embedded in a broader social context, and therefore, a stressor (like pediatric cancer) influences not only the development and adaptation of that child, but also the context in which s/he lives and the subsystems with which s/he interacts (Social Ecology Model: Bronfenbrenner, 1977; Cipolletta et al., 2015). Indeed, in the context of pediatric cancer, there is abundant empirical evidence for the impact of the diagnosis and its treatment on the parents, both at the level of their individual functioning and couple functioning.

Concerning the impact of pediatric cancer on *parents’ individual outcomes*, existing research revealed that a significant subset of parents report emotional distress, anxiety and acute or posttraumatic stress symptoms shortly after diagnosis (Grootenhuis and Last, 1997; Vrijmoet-Wiersma et al., 2008; Ljungman et al., 2014). Moreover, especially mothers seem to be impacted: they report more psychological distress than mothers of healthy children and fathers of children with cancer (Pai et al., 2007). In addition to the impact on parents’ individual functioning, many studies have documented the impact of pediatric cancer on *parents’ intimate relationship* (e.g., Hoekstra-Weebers et al., 1998; Patistea et al., 2000). A recently conducted systematic review (Van Schoors et al., 2017) revealed that although most couples adjust well to the crisis of a pediatric cancer diagnosis in domains such as emotional closeness, couple support and marital satisfaction, most couples do experience difficulties in the domains of sexual intimacy and conflict, both on and off treatment.

It should be noted, however, that the research described above also revealed a considerable variability -both across and within studies- in individual outcomes as well as relationship outcomes for parents facing pediatric cancer. Given this great variability, a growing number of studies has tried to explain why some parents adjust better than others. Among these studies investigating risk and protective factors for individual and relationship functioning of parents being confronted with pediatric cancer, especially individual characteristics (e.g., catastrophic thoughts in parents; Caes et al., 2014) and family characteristics (e.g., family support; Fuemmeler et al., 2003) have been the topic of investigation. In contrast, so-called couple factors –characteristics of the intimate relationship of the child’s parents– that may foster or inhibit parental individual and relationship outcomes gained less research attention. The current study aimed to address this gap by focusing on a couple-level variable that could be expected to moderate the impact of pediatric cancer on parents’ individual and relationship outcomes, namely, the extent to which parents deal with the stressor of pediatric cancer as a dyad (“dyadic coping;” see Bodenmann, 1995). Dyadic coping has been identified in the couple research literature as well as the stress and coping literature as playing a cardinal role in individual and relationship functioning within couples facing severe stressors (e.g., Kayser et al., 1999; Bodenmann, 2005).

“Dyadic coping” should be distinguished from other ways of coping with stress within intimate relationships, such as partners’ individual coping (e.g., LaMontagne et al., 2003; Garro, 2004; Wong and Heriot, 2008) and their attempts at seeking social support from friends or relatives (e.g., Fife et al., 1987). In particular, in situations where there is the crossover of individual stress from one partner to the other (e.g., work stress) or in cases of partners’ shared stress from common sources (e.g., stress related to pediatric cancer), a joint appraisal of the stressful situation is required, which triggers dyadic coping, in addition to partners’ individual coping. Within the dyadic coping literature, positive as well as more negative forms of coping as a dyad are described. Positive forms of dyadic coping include supportive dyadic coping (i.e., one partner assists the other in his/her coping efforts) and common dyadic coping (i.e., both partners participate in the coping process together). Negative forms of dyadic coping include hostile (i.e., support accompanied by distancing or sarcasm), ambivalent (i.e., support that is unwillingly) or superficial (i.e., support that is insincere) dyadic coping (Bodenmann, 1995, 1997, 2005).

Both theoretical and empirical arguments speak to the need of investigating (the role of) dyadic coping in the context of pediatric cancer. First, according to the Systemic Transactional Model (STM) of Stress and Coping in Couples, stressors always affect (directly or indirectly) both partners in an intimate relationship. This is true if the situation concerns primarily one partner – then his/her stress reactions and coping affects the other and turn into dyadic issues, representing the cross-over of stress and coping from one partner to the other (i.e., stressor of the self/partner) – and if the situation concerns both partners (i.e., shared stressors), both with regard to stress from daily hassles and more severe stressors (Bodenmann et al., 2016). So, stress *and* coping need to be understood as a systemic issue, a social process rooted in intimate relationships, with special attention to the interdependence and the mutual influence between romantic partners (Bodenmann et al., 2016). According to this theory, a pediatric cancer diagnosis needs to be considered as a shared and “dyadic stressor,” as it is indeed a stressful event or encounter that concerns both partners, either directly or indirectly (Bodenmann, 1995, 1997). Both parents are *directly* involved in their child’s illness, as shown by the finding that mostly one parent (temporarily) quits his/her job in order to accompany the diagnosed child day and night (Van Schoors et al., 2018) or by the parents’ individual emotional consequences described earlier (e.g., Pai et al., 2007). Also in line with this theory is that a dyadic stressor requires dyadic coping, conceptualized as the way couples cope with stress together in sharing appraisals of demands and planning together how to deal with the stressors. The importance of studying dyadic coping within the context of pediatric cancer can be derived from studies underscoring the positive role of coping-related activities, such as individual coping (e.g., Grootenhuis and Last, 1997) and social support (e.g., Fife et al., 1987) for the adjustment of parents and their ill child. Second, the importance of dyadic coping within the context of couples facing health and illness-related issues has been equally documented. For instance, the positive effect of dyadic coping on individual outcomes like *health* is largely documented (e.g., Berg and Upchurch, 2007; Meier et al., 2011), also in adult cancer studies (e.g., Kayser et al., 1999; Badr et al., 2008). Previous studies furthermore show robust and consistent associations between dyadic coping and relationship outcomes (Falconier et al., 2015). More specifically, a recent systematic review that focuses on couples coping with adult cancer illustrates that positive dyadic coping (i.e., supportive dyadic coping and common dyadic coping) improves relationship functioning, while negative dyadic coping impedes relationship functioning (Traa et al., 2015).

Taken together, based on theory (STM) and previous research on chronic illnesses in adulthood, we expect that dyadic coping may also be of importance in the context of pediatric cancer. More specifically, we expect that adequate dyadic coping (i.e., more supportive dyadic coping, more common dyadic coping, and less negative dyadic coping) is associated with better individual outcomes (i.e., less negative emotions: less stress, anxiety and depression, and lower levels of childhood illness-related parenting stress) and better relationship outcomes (i.e., higher marital and sexual adjustment) within parents being confronted with cancer in their child.

**Table 1:** Background characteristics of couples of children with Leukemia or non-Hodgkin lymphoma.

	Demographic variable		Men | Women
Parents	*N* (couples)		59
	Age, mean (*SD*)		40.5 (6.7) | 38.5 (6.2)
	Education, *n*	Primary school	1| 0
		High school	21 | 16
		Bachelor/Master	37 | 43
Ill child	N		59
	Sex, boys, *n*		36
	Age, mean (*SD*)		7.7 (5.1)
	Diagnosis, *n*	Acute lymphoblastic leukemia (ALL)	43
		Acute myeloid leukemia (AML)	3
		Non-Hodgkin Lymphoma	13
	Time since diagnosis in months (*SD*; Range)		6.9 (6.6; 0–20)

## Materials and Methods

### Participants

The sample consisted of 59 heterosexual couples; all biological parents of children diagnosed with leukemia or non-Hodgkin lymphoma. They were all Caucasian and living in the Flemish part of Belgium. Mothers’ mean age was 38.5 (Range 29–52); fathers’ mean age was 40.5 (Range 30–56). Time since diagnosis varied from 0 to 20 months (*M* = 6.9, *SD* = 6.6). Forty-three women and thirty-seven men had a Bachelor or Master degree. In eight families, the diagnosed child was the only child. The remaining families had either two (28 families), three (20 families) or four (3 families) children. More details on the sample are listed in Table 1. Ethical approval from the University Hospitals of Ghent, Brussels, Antwerp, and Leuven had been secured for the study and the appropriate written informed consent forms were obtained for all participants.

### Procedure

The present study is part of a larger study examining the impact of pediatric cancer on families, i.e., “UGhent Families and Childhood Cancer study.” For this large-scale study, children diagnosed with leukemia or non-Hodgkin lymphoma between the age of one and 18 years, their biological parents and any siblings were invited to take part in a survey study. Exclusion criteria were: (1) not speaking Dutch, (2) expression of a developmental disorder in the diagnosed child, and (3) relapse. Over a period of 3 years, 129 families participated; i.e., 65% of the eligible families. In 65 of these families, both parents filled out the questionnaires (50%), 59 of whom were married/co-habiting (91%) and 6 were divorced (9%). As this study focuses on the intimate relationship, the final sample only included the married or co-habiting couples (*N* = 59).

### Measures

#### Dyadic Coping

A short version of the Dyadic Coping Inventory (DCI; Bodenmann, 2008) was used to measure several forms of dyadic coping. The questionnaire consists of 17 items, grouped into 6 subscales: Supportive Dyadic Coping (e.g., “S/he makes me feel that s/he understands me and is committed to me”), Common Dyadic Coping (e.g., “We try to tackle the problem together and work together”), Negative Dyadic Coping (e.g., “S/he does not take my stress seriously”), Own Stress Communication (e.g., “When I feel overwrought, I show my partner that I feel bad and that I need his/her emotional support”), WE-Stress Appraisal and Individual Stress-Appraisal. In this study, only the subscales supportive dyadic coping, common dyadic coping and negative dyadic coping were included given our focus on dyadic coping. Response options for each item ranged from 1 to 5 (“very rarely” to “almost always”). Scores for each subscale were obtained by summing the relevant items. The DCI has good reliability and validity (Ledermann et al., 2010). In the present study, Cronbach’s alpha coefficients were 0.53/0.83 (supportive dyadic coping), 0.67/0.95 (common dyadic coping) and 0.75/0.70 (negative dyadic coping) for men and women, respectively. The low Cronbach’s alpha for the male supportive dyadic coping subscale could not be improved by dropping one or more items.

#### Depression, Anxiety, Stress

The Depression Anxiety Stress Scale (DASS-21; Lovibond and Lovibond, 1995) is a brief version of the 42-item DASS and consists of 21 items exploring negative emotions experienced over the last week. Participants rate the extent to which feelings of depression (e.g., “I felt that I had nothing to look forward to”), anxiety (e.g., “I experience trembling”) and stress (e.g., “I found it hard to wind down”) apply to them on a four-point scale from 0 (never) to 3 (almost always). Scores for depression, anxiety and stress were obtained by summing the relevant seven items. The DASS-21 proved to be reliable in both clinical and community samples (Antony et al., 1998). In the present study, Cronbach’s alpha coefficients were (for men and women, respectively) 0.88/0.91 for depression, 0.77/0.79 for anxiety and 0.85/0.89 for stress.

#### Childhood Illness-Related Parenting Stress

The Pediatric Inventory for Parents (PIP; Streisand et al., 2001) measures childhood illness-related parenting stress. The questionnaire consists of 42 items grouped into four domain scales indicating the type of stressors parents are experiencing related to caring for their ill child: (1) medical care (e.g., “helping my child with medical procedures”), (2) communication (e.g., “speaking with child about his/her illness”), (3) role functioning (e.g., “being unable to go to work/job”), and (4) emotional functioning (e.g., “feeling numb inside”). Given the overlap between the DASS-21 and the emotional functioning subscale, the latter subscale was not included. In addition, both the frequency over the last week and the level of difficulty of each item is assessed on a five-point scale (frequency: 1 = “never” to 5 = “very often;” difficulty: 1 = “not at all” to 5 = “extremely”). Frequency and difficulty scores are summed for each of the three domain scales; these scale scores are then summed into an overall total frequency score (PIP-F) and total difficulty score (PIP-D) with higher scores indicating greater frequency and difficulty of illness-related stress. The PIP has good reliability and validity (Streisand et al., 2001). In the present study, Cronbach’s alpha coefficients were 0.92/0.92 for the total frequency score and 0.91/0.90 total difficulty score, men and women, respectively.

#### Marital Adjustment

The Maudsley Marital Questionnaire (MMQ; Arrindell et al., 1983) evaluates the marital relationship in general (e.g., “How much are you committed to this marriage?”), the sexual relationship (e.g., “Are you satisfied with the present frequency of sexual intercourse’?”) and life in general (e.g., “Are you competent and successful at your job and your housework’?”). The questionnaire contains 20 items, each of which is rated on a 0 – 8 scale, with 0 representing the optimum response. A cutoff score >20 on the marital adjustment scale can be used to identify individuals who experience marital dissatisfaction (a level of marital dissatisfaction equal to the one reported by couples referred for marital counseling; Tuinman et al., 2005). In our study, 18 men and 19 women reported a score above 20 on the marital adjustment scale. When comparing the means on the MMQ marital adjustment scale of our study with a recent, Belgian, community sample (Hellemans, 2014), the current sample reported significantly higher levels of marital dissatisfaction (*D* = 3.88, *t* = 3.63, *p* < 0.001*).* The MMQ has good reliability and validity and the psychometric qualities of the Dutch version were also found to be satisfactory (Arrindell et al., 1983; Orathinkel et al., 2007). In the present study, only the two relationship subscales were taken into account, with a Cronbach’s alpha of 0.91/0.67 for marital adjustment and 0.84/0.89 for sexual adjustment, men and women, respectively. For both subscales, a higher score indicates more maladjustment.

### Data Analytic Strategy

**FIGURE 1:**
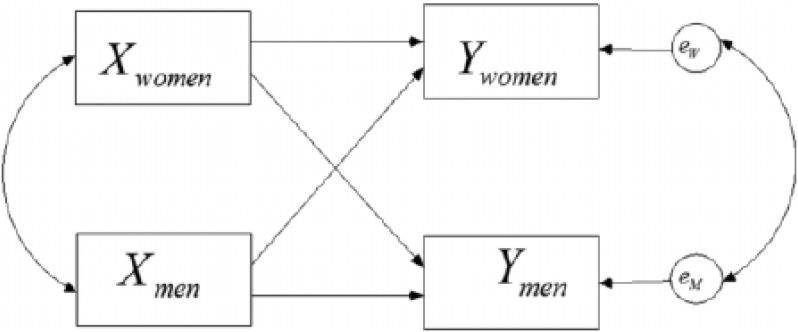
The Actor-Partner Interdependence Model (APIM). *X* represents one’ perception of dyadic coping (i.e., one of the dyadic coping subscales), while *Y* represents parenting stress (PIP), depression, anxiety and stress (DASS) or marital and sexual adjustment (MMQ subscales). For supportive and negative dyadic coping: an actor effect for women (men) can be interpreted as the effect of female (male) perception of her (his) partner’s supportive/negative coping efforts on the female (male) adjustment; a partner effect of women in men (of men in women) can be interpreted as the effect of female (male) perception of her (his) partner’s supportive/negative coping efforts on the partner’s adjustment. For common dyadic coping: an actor effect for women (men) can be interpreted as the effect of female (male) perception of the couple’s common coping efforts on the female (male) adjustment; a partner effect of women in men (of men in women) can be interpreted as the effect of female (male) perception of the couple’s common coping efforts on the partner’s adjustment.

We first describe means (with standard deviation and range) for all study variables and assess differences between men and women using a paired *t*-test. We further present correlations between study variables for men and women separately. The correlations for each study variable between men and women illustrate the non-independence within couples. To assess the association between the perception of supportive, common and negative dyadic coping (DCI) on the one hand and the frequency and difficulty of childhood illness-related parenting stress (PIP), depression, anxiety and stress (DASS), and marital and sexual adjustment (MMQ) on the other hand, we relied on the Actor-Partner Interdependence Model (APIM; Cook and Kenny, 2005). As shown in Figure 1, the APIM allows to simultaneously assess the effect of one’s own perception of dyadic coping and one’s partner perception of dyadic coping on one’s own (actor) and one’s partner outcome, while accounting for the correlation of outcomes within couples. The residuals of men and women were allowed to be correlated and to have a different variance (i.e., an unstructured residual covariance). A separate APIM was fitted for each combination of dyadic coping subscales and outcome allowing for differential effects for male and female partners. Only if the overall tests for actor and partner effects [that is, testing the goodness-of-fit of models without actor (partner) effects] turned significant, actor and partner effects were inspected. Note that in all analyses, the time since diagnosis was included as a covariate, and was allowed to have a different effect on the male and female outcomes. All analyses were performed in the Structural Equation Modeling (SEM) framework (Stas et al., 2018) using the R-package lavaan. Unstandardized regression coefficients for actor and partner effects are presented with corresponding standard error and *p*-value. To assess gender differences in actor and partner effects, the difference between the male and female actor effect (partner effect, respectively) was calculated (hereafter referred to as the difference test). All tests were performed at the 0.05 significance level. Given the exploratory nature of this study, no correction for multiple testing was performed.

**Table 2:** Descriptive statistics and correlations of the study variables.

Women			Men										
			1	2	3	4	5	6	7	8	9	10	11
			12.98 (2.27), 8–18	12.25 (2.26), 7–15	5.64 (2.30), 3–12	70.32 (17.89), 31–108	47.80 (14.49), 27–81	1.88 (2.47), 0–11	4.46 (4.19), 0–14	5.34 (3.65), 0–14	16.19 (11.90), 0–54	12.93 (8.93), 0–35	6.92 (6.57); 0–20
	1	**13.98^∗^ (3.25), 7–20**	0.062	0.412^∗∗^	−0.402^∗∗^	−0.008	0.051	−0.05	0.017	0.192	−0.292^∗^	−0.208	−0.210
	2	**12.22 (2.85), 4–15**	0.749^∗∗^	0.440^∗∗^	−0.578^∗∗^	−0.143	−0.347^∗∗^	−0.316^∗^	−0.319^∗^	−0.17	−603^∗∗^	−0.470^∗∗^	−0.265^∗^
	3	**5.93 (2.37), 3–11**	−0.658^∗∗^	−0.629^∗∗^	0.255	0.003	0.191	0.220	0.291^∗^	0.152	0.620^∗∗^	0.412^∗∗^	0.264^∗^
	4	**78.04^∗∗^ (21.07), 38–120**	0.009	0.056	0.120	0.377^∗∗^	0.610^∗∗^	0.095	0.380^∗∗^	0.380^∗∗^	−0.05	−0.011	−0.314^∗^
	5	**56.84^∗∗∗^ (17.81), 29–108**	0.060	0.073	0.036	0.643^∗∗^	0.459^∗∗^	0.376^∗∗^	0.513^∗∗^	0.663^∗∗^	0.146	0.231	0.001
	6	**3.73^∗∗∗^ (3.80), 0–18**	0.002	0.010	−0.028	0.414^∗∗^	0.504^∗∗^	0.284^∗^	0.591^∗∗^	0.622^∗∗^	0.32^∗^	0.318^∗^	0.212
	7	**6.07^∗^ (5.20), 0–21**	0.090	0.080	0.121	0.481^∗∗^	0.569^∗∗^	0.715^∗∗^	0.233	0.746^∗∗^	0.388^∗∗^	0.322^∗^	0.058
	8	**8.32^∗∗∗^ (4.70), 0–20**	0.062	0.017	−0.002	0.392^∗∗^	0.551^∗∗^	0.681^∗∗^	0.716^∗∗^	0.304^∗^	0.233	0.380^∗∗^	0.027
	9	**16.32 (11.42), 0–47**	−0.568^∗∗^	−0.707^∗∗^	0.639^∗∗^	−0.094	0.000	−0.093	−0.061	−0.034	0.709^∗∗^	0.744^∗∗^	0.359^∗∗^
	10	**12.05 (7.42), 0–32**	−0.227	−0.301^∗^	0.294^∗^	0.015	0.071	−0.084	0.103	0.097	0.468^∗∗^	0.762^∗∗^	0.262^∗^
	11	**6.92 (6.57), 0–20**	−0.107	−0.337^∗∗^	0.192	−0.576^∗∗^	−0.277^∗^	−0.245	−0.246	−0.227	0.307^∗^	0.058	1

*Bold = Mean (SD), Range with gender differences = ^∗^p < 0.05, ^∗∗^p < 0.01, and ^∗∗∗^p < 0.001. Underlined = inter gender correlations (between men and women). ^∗^Correlation is significant at the 0.05 level; ^∗∗^Correlation is significant at the 0.001 level. 1 = supportive dyadic coping (DCI): One’s perceptions of their partner’s supportive coping efforts; 2 = Common dyadic coping (DCI); 3 = Negative dyadic coping (DCI): One’s perceptions of their partner’s negative coping efforts; 4 = Parenting stress_Frequency (PIP-F); 5 = Parenting stress_Difficulty (PIP-D); 6 = Anxiety (DASS); 7 = Depression (DASS); 8 = Stress (DASS); 9 = Marital (Mal)adjustment (MMQ); 10 = Sexual (Mal)adjustment (MMQ), 11 = Time since Diagnosis. DCI, PIP, and DASS: higher scores indicate higher levels of coping, parenting stress and negative emotions; MMQ: higher scores indicate higher marital/sexual maladjustment.*

## Results

Table 2 shows the descriptive statistics and correlations of the variables in our study. For common and negative dyadic coping, no significant gender differences were found. However, women reported experiencing more supportive behavior (supportive dyadic coping) from their partner than their male partner (*D*^M−W^ = −1.00, *t*(56) = −2.03, *p* = 0.047). Furthermore, higher levels of childhood illness-related stress (frequency *D*^M−W^ = −7.72, *t*(57) = −2.82, *p* = 0.007; difficulty *D*^M−W^ = −9.04, *t*(57) = −4.45, *p* < 0.001), anxiety (*D*^M−W^ = −1.85, *t*(58) = −3.14, *p* < 0.001), depression (*D*^M−W^ = −1.61, *t*(58) = −2.11, *p* = 0.04) and stress (*D*^M−W^ = −2.98, *t*(58) = −4.59, *p* < 0.001) were found in women, as compared to men. Finally, regarding marital and sexual adjustment, no gender differences were found. Next, we discuss the results of the APIM-analyses (see Supplementary Table 1). We limit our discussion below to the gender-specific actor and partner effects for whom the global actor and partner test, respectively, were significant at 0.05 level (Table 3). Table 4 shows an overview of the significant APIM-results.

**Table 3:** APIM analyses.

	Overall Test	Difference Test
Actor effect CDC on PIP-D	*X*^2^(2) = 8.181, *p* = 0.017	*z* = −2.577, *p* = 0.010
Partner effect CDC on PIP-D	*X*^2^(2) = 9.223, *p* = 0.010	*z* = 3.120, *p* = 0.002
Partner effect SDC on PIP-D	*X*^2^(2) = 10.052, *p* = 0.007	*z* = 2.608, *p* = 0.009
Actor effect NDC on depression	*X*^2^(2) = 6.220, *p* = 0.045	*z* = 0.358, *p* = 0.720
Partner effect SDC on depression	*X*^2^(2) = 8.789, *p* = 0.012	*z* = 2.221, *p* = 0.026
Partner effect SDC on anxiety	*X*^2^(2) = 18.892, *p* < 0.001	*z* = 3.011, *p* = 0.003
Partner effect SDC on stress	*X*^2^(2) = 12.092, *p* = 0.002	*z* = 2.799, *p* = 0.005
Actor effect SDC on marital adjustment	*X*^2^(2) = 25.433, *p* < 0.001	*z* = 1.010, *p* = 0.312
Actor effect CDC on marital adjustment	*X*^2^(2) = 50.539, *p* < 0.001	*z* = 0.524, *p* = 0.600
Actor effect NDC on marital adjustment	*X*^2^(2) = 49.765, *p* < 0.001	*z* = −0.165, *p* = 0.869
Partner effect NDC on marital adjustment	*X*^2^(2) = 20.538, *p* < 0.001	*z* = 0.393, *p* = 0.694
Actor effect CDC on sexual adjustment	*X*^2^(2) = 14.308, *p* = 0.001	*z* = 1.406, *p* = 0.160
Actor effect NDC on sexual adjustment	*X*^2^(2) = 12.569, *p* = 0.002	*z* = 0.222, *p* = 0.824

*The Overall Test assesses whether the actor (partner, respectively) effects in both males and females are zero or not. The Difference Test assesses whether the actor (partner) effect is equal in men and women, or not. CDC = Common dyadic coping (DCI); PIP-D = Parenting stress_Difficulty (PIP-D); SDC = Supportive dyadic coping (DCI); NDC = Negative dyadic coping (DCI); depression, anxiety and stress (DASS); marital and sexual adjustment (MMQ).*

**Table 4:** APIM-results: an overview.

		Men							Women						
	
		1	2	3	4	5	6	7	1	2	3	4	5	6	7
Men	SDC						X			X	X	X	X		
	CDC		X				X	X		X					
	NDC			X			X	X						X	
Women	SDC													X	
	CDC													X	
	NDC						X							X	X

*SDC = supportive dyadic coping (DCI): One’s perceptions of their partner’s supportive coping efforts; CDC = Common dyadic coping (DCI); NDC = Negative dyadic coping (DCI): One’s perceptions of their partner’s negative coping efforts; 1 = Parenting stress_Frequency (PIP-F); 2 = Parenting stress_Difficulty (PIP-D); 3 = Depression (DASS); 4 = Anxiety (DASS); 5 = Stress (DASS); 6 = Marital (Mal)adjustment (MMQ); 7 = Sexual (Mal)adjustment (MMQ). X = Statistically significant effect.*

### Dyadic Coping and Individual Outcomes

#### Childhood Illness-Related Parenting Stress

More common dyadic coping reported by men was associated with lower difficulty scores of childhood illness-related parenting stress in men (actor effect; *B* = −2.58, *SE* = 0.89, *p* = 0.004). In addition, two partner effects were found: higher levels of supportive dyadic coping as perceived by men in their partner and more common dyadic coping reported by men were both associated with lower difficulty scores of parenting stress in women when facing illness in a child (*B* = −3.07, *SE* = 0.93, *p* = 0.001 and *B* = −3.14, *SE* = 1.09, *p* = 0.004; respectively).

#### Negative Emotions

When assessing the association between dyadic coping and negative emotions, one actor effect was found in men: higher levels of negative dyadic coping perceived by men were associated with higher levels of depression in men (*B* = 0.50, *SE* = 0.24, *p* = 0.034). Furthermore, 3 partner effects were present in women. Higher levels of supporting dyadic coping as perceived by men in their partner were associated with lower levels of depression in women (*B* = −0.82, *SE* = 0.27, *p* = 0.003), lower levels of anxiety in women (*B* = −0.78, *SE* = 0.19, *p* < 0.001) and lower levels of stress in women (*B* = −0.84, *SE* = 0.23, *p* < 0.001).

### Dyadic Coping and Relationship Outcomes

Both in men and women separately, actor effects of dyadic coping emerged when considering marital adjustment as outcome. In men, we found that higher levels of supportive dyadic coping as perceived by men in their partner and more common dyadic coping reported by men (*B* = −2.23, *SE* = 0.60, *p* < 0.001; *B* = −1.18, *SE* = 0.60, *p* = 0.050, respectively) were associated with higher levels of marital adjustment reported by men. Negative dyadic coping as perceived by men in their partner was found to be associated with lower levels of marital adjustment reported by men (*B* = 2.50, *SE* = 0.48, *p* < 0.001). In women, we found that higher levels of supportive dyadic coping as perceived by women in their partner and more common dyadic coping reported by women (*B* = −2.66, *SE* = 0.43, *p* < 0.001) were associated with higher levels of marital adjustment reported by women (*B* = −1.91, *SE* = 0.37, *p* < 0.001). Negative dyadic coping as perceived by women in their partner was found to be associated with lower levels of marital adjustment reported by women (*B* = 2.62, *SE* = 0.47, *p* < 0.001).

One partner effect was found for coping reported by women on relationship adjustment reported by men: lower levels of negative dyadic coping as perceived by women in their partner (*B* = 1.75, *SE* = 0.47, *p* < 0.001 for respectively) were associated with higher levels of relationship adjustment as reported by their partner. Furthermore, negative dyadic coping as perceived by men in their partner was associated with lower levels of relationship adjustment reported by women (*B* = 1.37, *SE* = 0.48, *p* = 0.005).

When considering sexual adjustment as an outcome, only actor effects were observed in men and women for some dyadic coping subscales. More specifically, higher levels of common dyadic coping reported by men was associated with higher levels of sexual adjustment reported by men (*B* = −1.60, *SE* = 0.53, *p* = 0.003). Furthermore, for both men and women, higher levels of perceived negative dyadic coping in the partner were linked to lower levels of sexual adjustment (actor effects; *B* = 1.29, *SE* = 0.47, *p* = 0.006 for men and *B* = 0.83, *SE* = 0.41, *p* = 0.046 women, respectively).

## Discussion

Using an Actor-Partner Interdependence Model (APIM; Cook and Kenny, 2005), the present study sought to examine whether dyadic coping was related to individual outcomes (negative emotions: anxiety, depression & stress and childhood illness-related parenting stress) and relationship outcomes (marital adjustment and sexual adjustment) in parents of children diagnosed with blood cancer.

### Summary of Results

#### Dyadic Coping and Individual Outcomes

Our findings indicate that both *positive* (i.e., supportive and common dyadic coping) and negative forms of dyadic coping matter for individual outcomes within parents being confronted with a cancer diagnosis in their child. This is in line with our prediction and with previous quantitative research on adult chronic illnesses (e.g., Meier et al., 2011; Regan et al., 2014). However, different patterns of findings emerged for supportive, common and negative dyadic coping.

More specifically, we found that the more men perceived their partner as *supportive*, the less depression, anxiety and stress (both general stress and difficulty scores on childhood illness-related stress) their partner experienced. In other words, the more men perceived their spouse as supportive, understanding and helping, the better the female *partner’s* individual adjustment when facing pediatric cancer. These associations are in line with existing evidence that couple support is a protective and helpful factor in the individual adjustment to pediatric cancer (e.g., Morrow et al., 1982; Tarr and Pickler, 1999). However, we did not find the expected actor effects; i.e., associations between perceived supportive dyadic coping in one’s partner and one’s own individual adjustment. These findings seem to suggest that the benefits of support are mostly associated with support *giving* rather than support receiving, a finding that has also been reported by other researchers in the context of health outcomes (Brown et al., 2003). Furthermore, more *common dyadic coping* reported by men was associated with lower difficulty scores on illness-related parenting stress for men and for women. So, the more men had the experience that both partners participated in the coping process symmetrically or complementary, the less they and their partner struggled with the care of their ill child.

Finally, the more men perceived their partner as negative, the more depressive complaints they experienced. This is in line with previous studies investigating the association between negative dyadic coping and negative emotions in adult chronically ill populations (e.g., Meier et al., 2011). Looking at the differential effects of the different types of dyadic coping, negative dyadic coping seems to be of less importance for the individual well-being of parents facing pediatric cancer. This finding is not in line with the literature on adult chronic illness describing negative forms of dyadic coping to be frequently occurring (Meier et al., 2011). This contradiction can be understood in two possible ways. First, it is possible that partner effects between negative dyadic coping and individual adjustment were not found in this study due to the relative small sample size (mimicking the observed associations, the power to detect such effects with *N* = 59 couples ranged from 5 to 64%). Second, in the context of adult chronic illness, there is one partner undergoing the illness, and one experiencing the illness from a certain distance. In the context of a child’s cancer diagnosis, however, the child is ill, and therefore both parents may experience the illness in a more similar way. As a consequence, it is possible that couples, after facing a cancer diagnosis in their child, tend to understand each other better than in the context of adult chronic illness, and therefore, possibly engage less in negative dyadic coping.

#### Dyadic Coping and Relational Outcomes

For relationship outcomes within parents being confronted with a cancer diagnosis in their child, our findings indicated that both positive (i.e., supportive and common dyadic coping) and negative forms of dyadic coping matter. This is in line with our prediction, published quantitative studies (e.g., Falconier et al., 2015) and a recent systematic review (Traa et al., 2015) in the context of adult chronic illnesses. More specifically, the present study shows that positive dyadic coping (i.e., supportive and common dyadic coping) was associated with higher marital adjustment, both in men and women (actor effects). In other words, the more a man perceives his partner as supportive and helping and the more he has the idea that both partners participate in the coping process symmetrically or complementary, the more he is satisfied with his marital relationship. The same pattern of findings was found for women. Furthermore, for negative dyadic coping, the more a man experiences distancing, mocking or sarcasm in his partner when talking about the illness, the less satisfied he is with his marital relationship. Again, this finding was replicated in women. Next to these so-called actor effects, the following partner effects were also found for marital adjustment. The more men and women perceived their partner as negative when talking about the cancer, the less satisfied their partner was in the marital relationship.

With regard to sexual adjustment, the more men experienced managing the cancer situation together, and the less negative their partner reacts, the more satisfied they were with their sexual relationship. Furthermore, the more women perceived their partner as negative, hostile or not interested, the less satisfied they were with their sexual relationship. These findings extend existing research by demonstrating that dyadic coping is not only related to marital adjustment and marital satisfaction (e.g., Falconier et al., 2015) but also to couples’ sexual satisfaction.

Remarkably, for relationship adjustment, both actor effects and certain partner effects of dyadic coping were found to be important, whereas for sexual adjustment, only actor effects proved to be significant. So, how a parent describes the way in which s/he and his/her partner, as a couple, cope with the stressor together (i.e., supportive, negative or common) was at least partially related to their own and their partner’s evaluation of the relationship (actor and partner effect) but only to their own evaluation of the sexual relationship (actor effect). The absence of partner effects in explaining sexual adjustment may be linked to the fact that sexuality is, in se, an intimate domain and a difficult topic to discuss. As a consequence, the assessment of one’s sexual relationship may be primarily linked to one’s own appraisal of dyadic coping.

#### Gender

Gender differences as well as important gender similarities emerged from our data. Although at the relationship level, men and women reported to be equally satisfied with their partner and their sexual relationship, men and women did differ with regard to their individual adjustment. Across all individual outcomes, women reported higher levels of maladjustment (i.e., child’s illness-related stress, anxiety, depression, stress) when facing a cancer diagnosis in their child than their male partner. This is in line with previous studies in the context of pediatric cancer, showing that especially mothers are impacted by the illness of the child (Pai et al., 2007). This finding may be explained by the increased burden assumed to be experienced by mothers in the care of children with cancer, as they are for example more likely to accompany the child to medical procedures (Kazak et al., 1996) and to stay in the hospital day and night (Van Schoors et al., 2018). In terms of dyadic coping, men and women only seemed to differ in the amount of supportive coping they perceived in their partner, with women reporting higher levels of supportive coping in their partner than men. These findings are not in line with the so-called marital support gap hypothesis, assuming that women are better support providers in their relationship than men are (see Verhofstadt et al., 2007 for a critical discussion). Comparing this finding to existing research on gender differences and similarities in dyadic coping is hard, however, as previous research focused on populations in which one of the partners was ill and therefore in a more support seeking/receiving position. Furthermore, important similarities between men and women in the association between dyadic coping and the relational outcomes under study emerged, more specifically the actor effects of dyadic coping on marital adjustment. Indeed, no significant differences were found in the actor/partner effects on relational outcomes between males and females (Table 3). This means that the pattern of findings found in our male subsample was fully replicated within our female subsample and that for both parents of children with cancer, dyadic coping and relationship functioning are intertwined. However, the absence of evidence for a difference might also be due to the low power to detect such interactions in small samples (Gistelinck et al., 2018). For the individual outcomes, the patterns for men versus women were more heterogeneous, thus less parallels could be drawn between them. Indeed, several of the observed actor and partner effects on individual outcomes were significantly different between men and women (Table 3). Finally, gender effects also emerged in terms of effects of the predictor (i.e., the perception of dyadic coping). More specifically, men and women only differed in the *partner effects* of supportive dyadic coping on the individual outcomes (i.e., anxiety, depression and stress), and not in the actor effects. For common dyadic coping, however, gender differences were found in both actor and partner effects (Table 3). These tentative findings deserve further exploration in future research.

It is important to note that since no Type-I error correction was performed in this exploratory study, caution is warranted with regard to the interpretation of the above findings. All these findings should be reproduced in future studies.

### Strengths and Limitations

A strength of this study is that it is the first to explore the association between dyadic coping and parental adjustment (individual and relationship outcomes), both within and between partners, after being confronted with a cancer diagnosis in their child. Furthermore, although most studies in the childhood cancer literature make use of a single-family member participant (e.g., Van Schoors et al., 2015), we included the perspectives of both partners. Discrepancies in perceptions across family members/partners (e.g., Alderfer et al., 2009) speak to the need to collect data from both members (e.g., Van Schoors et al., 2018). Additionally, by making use of the Actor-Partner Interdependence Model (APIM; Cook and Kenny, 2005), we were able to model the interdependence in the dyadic relationship.

Despite the strengths of this study, some important limitations should be noted. First, we used a sample of Caucasian, heterosexual couples, thereby limiting the generalizability of our results. Future research should attempt to replicate these findings with more heterogeneous samples, e.g., also homosexual couples. Second, only Dutch speaking parents were included for participation. Therefore, with respect to the current multicultural society, this language criterion might have been a barrier for ethnic minorities. Third, we only focused on children with leukemia or non-Hodgkin lymphoma. As a consequence, it is important to highlight that parents of children with other cancer diagnoses may have different experiences. Fourth, time since diagnosis varied between the couples, ranging from 0 to 20 months. The potential biases inherent in retrospective methods like the one used in the current paper may have influenced their responses (e.g., forgetting, defensiveness). In addition, future (longitudinal) studies should also take into account the possible impact of time since diagnosis, as it is plausible to assume that the effect of dyadic coping on outcomes has a different impact depending on how long the parents face the illness of their child. Now, we simply adjusted for the effect of time since diagnosis on the outcomes, but future studies may look at the interaction of time since diagnosis and the actor and partner effects of dyadic coping. Fifth, as the associations described in this study are correlational in nature, the temporal order of the variables under investigation could not be tested with the present data. It is also possible, for instance, that better parental adjustment elicits more adaptive dyadic coping strategies, as described above.

### Clinical Implications

Difficulties in the couple relationship may seem secondary to the more pressing need of ensuring adequate cancer and psychosocial care for the child. Therefore, such issues may be overlooked by psychosocial care providers in oncology or may even be downplayed by the couples themselves. However, this study shows that dyadic coping matters for individual and relational functioning in parents when facing cancer in their child. As a consequence, it is important to screen and tackle relational issues besides individual issues, taking into account evidence-based standards for psychosocial care in pediatric oncology. Interventions aimed at dealing with couple problems that get in the way of cancer care or hamper the adjustment of the child and/or family should take into account two specific recommendations. First, in working with families being confronted with a cancer diagnosis in a child, clinicians should not only focus on the adjustment of the child diagnosed with cancer or educational issues that arise post-diagnosis, but also on the impact of the illness on the parents in general and the parents’ intimate relationship in particular. Moreover, clinicians should invite the couple system as a whole. Only by taking into account the perspectives of both members, couple level variables – such as dyadic coping – can be fully understood and improved when needed. Second, as previous research demonstrated that sexual relationships appear to be affected most negatively when facing a cancer diagnosis in their child (Lavee and May-Dan, 2003), clinicians should overcome their potential reluctance to discuss such topics together with the couple. Third, clinical interventions should be tailored to gender differences and specific characteristics of men and women facing pediatric cancer. For example, our findings suggest that women might be more vulnerable than men (cf. women reporting higher levels of individual maladjustment compared to men) when facing cancer in their child, and might therefore be in greater need of professional support from psycho-social workers or clinicians.

## Conclusion

Taken together, these findings led us to the conclusion that both positive and negative dyadic coping are important for individual as well as relationship outcomes of parents when facing a diagnosis of cancer in their child. Moreover, differential associations seem to be at play between different types of coping on the one hand and individual and relationship adjustment on the other hand. In addition, while men and women reported to be equally satisfied with their partner and their sexual relationship, women reported higher levels of individual maladjustment.

## Author Contributions

All authors had been seen and reviewed the manuscript, and contributed to it in a meaningful way. MVS wrote the manuscript under the supervision of LG and LV. TL did the analyses. GB, BO, JL, and KN helped in particular with the clinical implications of the manuscript.

## Conflict of Interest Statement

The authors declare that the research was conducted in the absence of any commercial or financial relationships that could be construed as a potential conflict of interest.
