# Supplementary material for: Couples Dealing With Pediatric Blood Cancer: A Study on the Role of Dyadic Coping
Source: Front Psychol. 2019 Feb 27;10:402. doi: 10.3389/fpsyg.2019.00402 (PMC6400881; doi:10.3389/fpsyg.2019.00402)
Supplement: Supplementary file 1 [file Table_1.docx]

|  |  | SDC | CDC | NDC |
| --- | --- | --- | --- | --- |
| PIP_F |  |  |  |  |
| Actor Effect | M 🡪 M | -0.467 (0.940) | -2.305 (1.105) * | 0.732 (0.977) |
|  | F 🡪 F | -0.302 (0.693) | -0.582 (0.882) | 2.175 (0.966) * |
|  | D | -0.165 (1.174) | -1.723 (1.471) | -1.443 (1.412) |
| Partner Effect | F 🡪 M | 1.081 (0.653) | 1.208 (0.865) | -0.598 (0.943) |
|  | M 🡪 F | -1.641 (0.998) | -1.858 (1.127) | -0.153 (1.001) |
|  | D | 2.722 (1.198) * | 3.066 (1.477) * | -0.444 (1.413) |
| Time Effect | M | -0.904 (0.326) ** | -0.910 (0.346) ** | -0.952 (0.337) ** |
|  | F | -1.981 (0.346) *** | -2.074 (0.353) *** | -1.982 (0.345) *** |
| PIP_D |  |  |  |  |
| Actor Effect | M 🡪 M | 0.376 (0.828) | -2.583 (0.886) ** | 1.145 (0.832) |
|  | F 🡪 F | 0.259 (0.649) | 0.821 (0.851) | 0.337 (0.975) |
|  | D | 0.117 (1.064) | -3.403 (1.320) ** | 0.808 (1.341) |
| Partner Effect | F 🡪 M | -0.183 (0.575) | 1.156 (0.694) | 0.182 (0.803) |
|  | M 🡪 F | -3.074 (0.934) ** | -3.142 (1.088) ** | 1.745 (1.011) |
|  | D | 2.890 (1.108) ** | 4.297 (1.377) ** | -1.563 (1.350) |
| Time Effect | M | -0.054 (0.287) | -0.105 (0.278) | -0.187 (0.287) |
|  | F | -0.957 (0.324) ** | -0.883 (0.341)** | -0.930 (0.349) ** |
| DASS DEPRESSION |  |  |  |  |
| Actor Effect | M 🡪 M | 0.047 (0.242) | -0.392 (0.265) | 0.504 (0.237) * |
|  | F 🡪 F | 0.121 (0.189) | 0.118 (0.259) | 0.368 (0.284) |
|  | D | -0.074 (0.308) | -0.511 (0.383) | 0.135 (0.377) |
| Partner Effect | F 🡪 M | -0.104 (0.168) | -0.214 (0.207) | 0.065 (0.229) |
|  | M 🡪 F | -0.818 (0.272) ** | -0.467 (0.331) | 0.118 (0.294) |
|  | D | 0.714 (0.321) * | 0.253 (0.403) | -0.053 (0.380) |
| Time Effect | M | 0.018 (0.084) | -0.043 (0.083) | -0.030 (0.082) |
|  | F | -0.268 (0.094) ** | -0.234 (0.104) * | -0.252 (0.102) * |
| DASS ANXIETY |  |  |  |  |
| Actor Effect | M 🡪 M | -0.018 (0.123) | -0.062 (0.139) | 0.182 (0.127) |
|  | F 🡪 F | -0.010 (0.129) | -0.055 (0.188) | 0.021 (0.208) |
|  | D | -0.008 (0.179) | -0.007 (0.244) | 0.161 (0.250) |
| Partner Effect | F 🡪 M | -0.161 (0.085) | -0.204 (0.109) | -0.077 (0.123) |
|  | M 🡪 F | -0.777 (0.185) *** | -0.227 (0.240) | 0.101 (0.216) |
|  | D | 0.617 (0.205) ** | 0.023 (0.272) | -0.177 (0.254) |
| Time Effect | M | 0.053 (0.043) | 0.027 (0.044) | 0.051 (0.044) |
|  | F | -0.214 (0.064) ** | -0.185 (0.075) * | -0.168 (0.074)* |
| DASS STRESS |  |  |  |  |
| Actor Effect | M 🡪 M | 0.331 (0.203) | -0.066 (0.236) | 0.225 (0.211) |
|  | F 🡪 F | 0.070 (0.162) | -0.088 (0.230) | 0.094 (0.255) |
|  | D | 0.260 (0.262) | 0.021 (0.344) | 0.131 (0.339) |
| Partner Effect | F 🡪 M | -0.075 (0.141) | -0.149 (0.184) | -0.016 (0.203) |
|  | M 🡪 F | -0.844 (0.234) *** | -0.152 (0.294) | 0.021 (0.264) |
|  | D | 0.0769 (0.275) ** | 0.003 (0.361) | -0.038 (0.341) |
| Time Effect | M | 0.017 (0.070) | -0.030 (0.074) | -0.022 (0.073) |
|  | F | -0.243 (0.081) ** | -0.211 (0.092) * | -0.194 (0.091) * |
| MMQ RELATION |  |  |  |  |
| Actor Effect | M 🡪 M | -1.183 (0.604)* | -2.231 (0.601) *** | 2.504 (0.484) *** |
|  | F 🡪 F | -1.907 (0.369) *** | -2.659 (0.428) *** | 2.620 (0.466) *** |
|  | D | 0.724 (0.716) | 0.428 (0.817) | -0.117 (0.706) |
| Partner Effect | F 🡪 M | -0.875 (0.420) * | -1.046 (0.470) * | 1.647 (0.467) *** |
|  | M 🡪 F | -0.097 (0.531) | -0.294 (0.547) | 1.370 (0.483) ** |
|  | D | -0.778 (0.686) | -0.752 (0.802) | 0.277 (0.706) |
| Time Effect | M | 0.476 (0.209) * | 0.274 (0.188) | 0.269 (0.167) |
|  | F | 0.421 (0.184) * | 0.108 (0.171) | 0.225 (0.167) |
| MMQ SEXUAL |  |  |  |  |
| Actor Effect | M 🡪 M | -0.694 (0.491) | -1.600 (0.533) ** | 1.288 (0.473) ** |
|  | F 🡪 F | -0.509 (0.299) | -0.567 (0.368) | 0.827 (0.414) * |
|  | D | -0.185 (0.583) | -1.033 (0.735) | 0.461 (0.680) |
| Partner Effect | F 🡪 M | -0.441 (0.341) | -0.168 (0.417) | 0.614 (0.456) |
|  | M 🡪 F | -0.222 (0.431) | -0.831 (0.470) | 0.463 (0.429) |
|  | D | -0.219 (0.558) | 0.663 (0.718) | 0.151 (0.678) |
| Time Effect | M | 0.271 (0.170) | 0.182 (0.167) | 0.186 (0.163) |
|  | F | 0.033 (0.149) | -0.073 (0.147) | -0.023 (0.148) |

**Supplementary Table 1. APIM-analyses.**

*NOTE*: The table presents estimated regression coefficients (with standard error in brackets) for the actor and partner effect for males (M) and females (F), and the difference in actor (resp. partner) effects between males and females (D); and the estimated regression effects for the effect of the time since diagnosis in men and women. A separate APIM was fitted for each combination of dyadic coping subscale and outcome.
